# Varicoceles in Men With Non-obstructive Azoospermia: The Dilemma to Operate or Not

**DOI:** 10.3389/frph.2022.811487

**Published:** 2022-04-04

**Authors:** Aris Kaltsas, Eleftheria Markou, Athanasios Zachariou, Fotios Dimitriadis, Charalampos Mamoulakis, Sotirios Andreadakis, Ioannis Giannakis, Panagiota Tsounapi, Atsushi Takenaka, Nikolaos Sofikitis

**Affiliations:** ^1^Laboratory of Spermatology, Department of Urology, Faculty of Medicine, School of Health Sciences, University of Ioannina, Ioannina, Greece; ^2^Department of Urology, School of Medicine, Aristotle University of Thessaloniki, Thessaloniki, Greece; ^3^Department of Urology, Medical School, University of Crete, Heraklion, Greece; ^4^Department of Urology, School of Medicine, Tottori University, Yonago, Japan

**Keywords:** non-obstructive azoospermia, varicocele, varicocelectomy, ICSI, spermatozoa

## Abstract

The knowledge on male reproduction is constantly expanding, especially in treating infertility due to non-obstructive azoospermia (NOA). Varicocele is occasionally diagnosed in a subpopulation of males with NOA. Varicocele repair in NOA-men may contribute to the reappearance of spermatozoa in semen. However, spontaneous pregnancies are observed in only a small percentage of NOA-men post-varicocelectomy. Additionally, it has been reported that the repair of varicocele in NOA-men (before the performance of sperm retrieval techniques) may increase the testicular sperm recovery rate. In addition, it increases the pregnancy rate in intracytoplasmic sperm injection (ICSI) programs in NOA-men without spermatozoa in the semen post-varicocelectomy. In addition, to the improvement in Sertoli cellular secretory function, varicocelectomy may increase the secretory function of Leydig cells, which subsequently results in improved androgen production, raising the probability to negate the need for testosterone replacement therapy in cases of late-onset hypogonadism. On the other hand, the benefit of varicocelectomy in patients with NOA is still debatable. The current review study aims to provide a critical and extensive review of varicocele repair in males with NOA. This study additionally focuses on the impact of varicocele repair on sperm retrieval rates and its influence on the ICSI outcomes for those couples who remain negative for spermatozoa in their semen samples post-varicocelectomy.

## Introduction

Male infertility treatment has significantly progressed recently. The intracytoplasmic sperm injection (ICSI) allowed overcoming difficulties in cases of a small number of functional spermatozoa in semen samples (1). Non-obstructive azoospermia (NOA) is one of the most challenging causes of male infertility to treat. It is described as the absence of spermatozoa in the microscopic evaluation of the semen sample observing the pellets of two semen samples post-centrifugation (2, 3).

Azoospermia affects 1% of the male population and 10–15% of a subpopulation of infertile men. Azoospermia is classified into obstructive azoospermia and NOA. Obstructive azoospermia is caused by an obstruction in any location in the seminal tract (2). On the other hand, NOA is attributed to a failure of spermatogenesis due to several pathophysiologies such as acquired lesions (i.e., exposure to environmental toxins, taking gonadotoxic medications, trauma, hormone imbalances, varicocele; among others) or genetic disorders (i.e., Y chromosome deletion, karyotype abnormalities, X-linked genes associated with male infertility; among others), and idiopathic disorders. Varicocele is a dilation of either the pampiniform plexus or the cremasteric plexus of the testis. NOA-men with palpable varicoceles represent a percentage equal to 4–14% of the population of NOA-men (4, 5).

For the first time, a spontaneous pregnancy has been reported after surgical repair of varicocele in a couple with NOA in 1952 by Tulloch (6). The beneficial effects of varicocelectomy in NOA-men occasionally represent an improvement in spermatogenesis and the reappearance of motile spermatozoa in the ejaculate. The overall result is that couples may achieve spontaneous pregnancy without ICSI procedures and thus may avoid the need for sperm retrieval techniques from testes (7, 8).

During the last years, the impact of varicocelectomy in NOA-men with palpable varicocele on semen parameters, testicular sperm retrieval rate, and pregnancy rate, have been extensively studied. Also, research efforts demonstrated predictors of (a) reappearance of spermatozoa in the ejaculate and (b) testicular sperm recovery in NOA-men after varicocelectomy (8–11).

In the current review study, we will attempt to review and evaluate the evidence provided by the international literature concerning whether the performance of varicocelectomy in NOA-males (a) may lead to the appearance of spermatozoa in the ejaculate, or (b) may result in higher testicular sperm recovery rates post-testicular sperm extraction (TESE); or (c) may be accompanied by higher pregnancy rates or live birth rates post-TESE.

## Effects of Varicocele and Varicocelectomy on Sertoli- and Leydig Cellular Function in Non-Obstructed Azoospermic Men Influencing the Progression of Spermatogenesis

The development of left varicocele in humans increases testicular temperature (12). An increase in testicular temperature in humans is known to detrimentally affect the Leydig cellular secretory function (12–17). In fact, a subpopulation of males with left varicocele demonstrates low peripheral serum levels of testosterone (18). The first report that described improved testosterone levels after varicocelectomy was published in 1975 by Comhaire and Vermeulen (19). The authors reported that testosterone profiles in infertile males increase after varicocele repair (19). It is known that androgens have an important role in (a) the regulation of the Sertoli cellular secretory function that is important for the induction of male meiosis (20), and (b) the completion of the spermiogenesis process (21). Therefore, for the subpopulation of males in whom varicocele has a severely detrimental effect on Leydig cell secretory function, the final result may be the inability of the male gamete to undergo meiosis or the inability of the early haploid male gamete to undergo elongation. In both cases, the phenotype will be azoospermia. This hypothesis is strongly supported by Cozzolino and Lipshults (22) stating that a varicocele may cause a progressive harmful effect on the testes that may finally lead to irreversible infertility if left without therapeutic management. The decrease in Leydig cellular secretory function may cause Sertoli cellular secretory function in men with varicoceles since, as we have already mentioned, the androgen stimulation of Sertoli cells is of paramount importance for their function (23). Thus males with varicoceles may demonstrate variable degrees of Sertoli cellular secretory dysfunction, occasionally resulting in azoospermia (24). Furthermore, the increase in testicular temperature may directly affect detrimentally the Sertoli cellular structure or function (25). In fact, induced increases in testicular temperature have raised the role of heating as a method of contraception.

**Figure 1:**
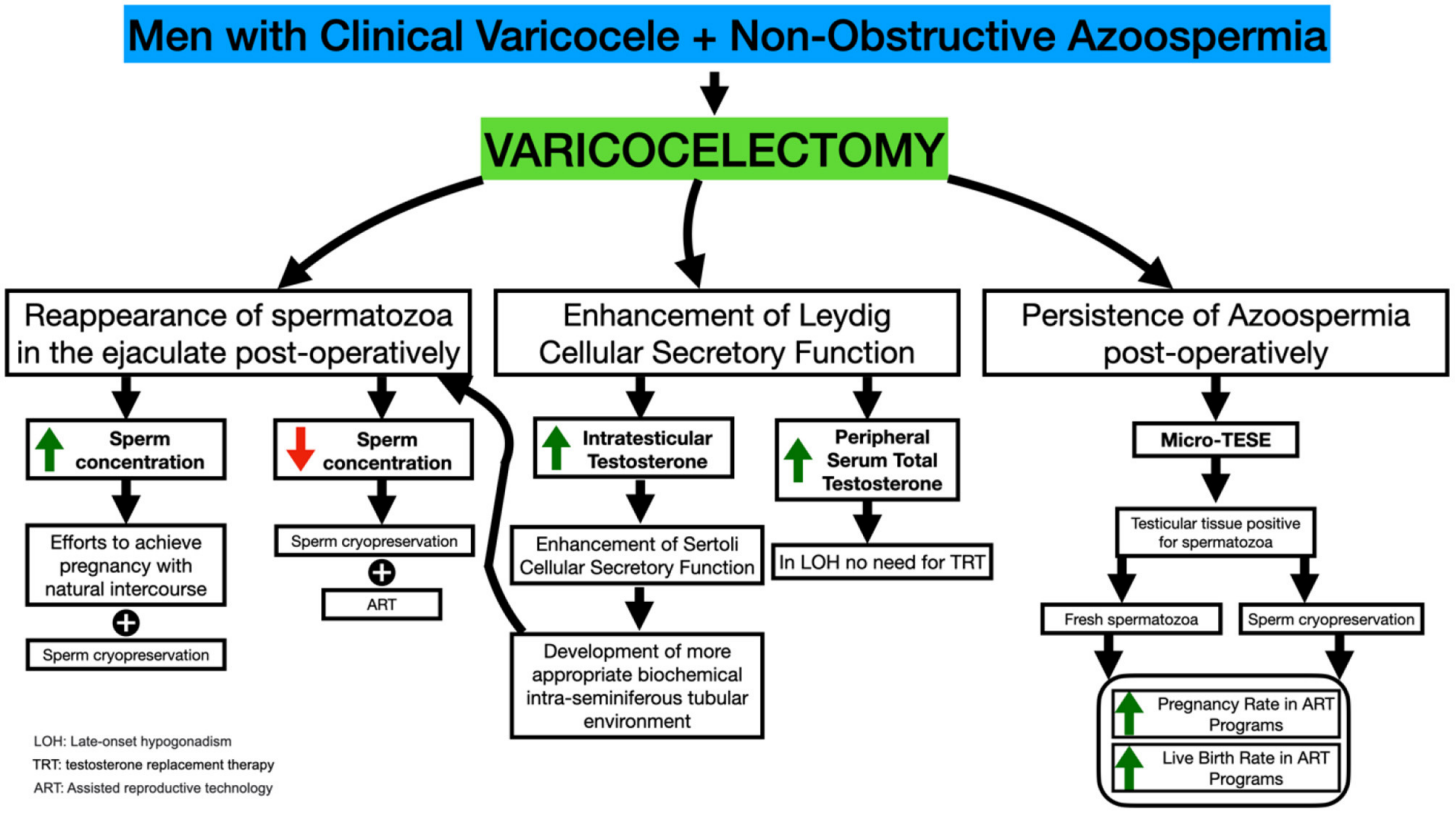
Varicocelectomy in NOA-men: mechanisms modulating the appearance of spermatozoa in the semen, SRRs and ICSI outcome.

Thus, severe Sertoli cellular dysfunction leading to NOA may occur in a subpopulation of males with varicocele (Figure 1). It has been suggested (26) that a high grade varicocele may induce sufficient testicular damage to result in the most severe testicular histological architecture associated with NOA and Sertoli cell-only (SCO) syndrome. Furthermore, testicular hypoxia attributable to impairment in venous drainage of the male genital system has been suggested to occur in males with varicoceles (27). This finding, taken together with the fact that SCO syndrome may be related in some individuals to persistent, longstanding male gonad hypoxia, may allow us to suggest that varicocele leads to an SCO syndrome in a subpopulation of patients (26).

The above paragraph indicates that varicocele development in humans may result in primary testicular damage and occasionally in severe secretory dysfunction of the Leydig and Sertoli cellular subpopulations that may contribute to the development of the azoospermic phenotype. On the other hand, Wright (28) has demonstrated the efficacy of varicocelectomy in restoring testicular temperature in humans. The latter study, taken together with the conclusions of the studies by Zorgnioti (29, 30), indicating that chronic scrotal hypothermia may be a treatment for poor semen quality, allows us to suggest that performance of varicocelectomy may alter cases of severe spermatogenic arrest to an oligospermic phenotype attributable to a decrease in intratesticular temperature. Furthermore, varicocelectomy has been accompanied by an increase in Leydig cellular secretory function resulting in increased peripheral serum testosterone profiles (31–36) and subsequently a more optimal stimulation of the Sertoli cellular secretory function. The overall result a possible alteration of the azoospermic phenotype to an oligospermic phenotype. Furthermore, the performance of varicocelectomy has been proven to improve Sertoli cellular secretory function (i.e., markers of Sertoli cell Secretory function) (37).

Furthermore, a study by Mubasher et al. (38) indicates high production of reactive oxygen species in a number of azoospermic subjects with varicoceles. Varicocelectomy has the potential to diminish the reactive oxygen species generation and up-regulates the antioxidant activity of seminal plasma (39, 40). Therefore, it may be suggested that varicocelectomy reducing the production of reactive species may turn the phenotype of azoospermia to an oligozoospermic phenotype. The latter suggestion is further enforced by a previous report indicating very vividly that oxidative stress has a detrimental effect on Sertoli cell function (41).

## Preoperative Predictors of Appearance of Spermatozoa in Semen Samples in NOA-Men After Varicocelectomy

### Histology

Various prognostic factors have been evaluated to identify patients who are more likely to demonstrate a beneficial effect on sperm parameters post-varicocelectomy. Aboutaleb et al. (42) attempted to evaluate the role of testicular biopsy histopathology to identify a subpopulation of NOA-men who will demonstrate restoration of spermatogenesis after subinguinal varicocelectomy in NOA-men. In that study (20), NOA-men with palpable bilateral varicocele underwent subinguinal varicocele repair and testicular biopsy at once. Spermatogenesis was restored in six men (30% of the male population of NOA-men). The outcomes of the testicular biopsy revealed the following histopathological patterns: SCO syndrome in 10 NOA-men, maturation arrest in 3 men, and hypospermatogenesis in 7 men. The restoration of spermatogenesis led to the reappearance of the sperm in the ejaculates (from 3 million to 15 million/ml) in the hypospermatogenesis-patients only (6 patients). On the other hand, patients with a histopathological pattern of maturation arrest (at the primary spermatocyte stage) or SCO syndrome did not show any significant change in semen analysis after varicocelectomy (42).

Moreover, Majzoub et al. (43) reported that motile spermatozoa in the ejaculate were observed in 11 (26.2%) of 42 NOA-men who had undergone varicocelectomy. Testicular histopathology only has a significant predictive value to predict the outcome of TESE patients who have undergone varicocelectomy, among other factors investigated. Post-varicocelectomy, (a) eight out of 11 (72.7%) patients who exhibited hypospermatogenesis pre-varicocelectomy, and (b) three out of 11 (27.3) patients with SCO regained sperm in the ejaculate (43).

A meta-analysis comparing the results of varicocelectomy in NOA-men, based on diagnostic testicular biopsy profiles, showed that there is a greater chance of successful induction of the spermatogenesis in NOA-men with maturation arrest or hypospermatogenesis compared to men with SCO (10). Percentages of males who demonstrated spermatozoa in their semen samples post-varicocele repair were 35.3% in the subpopulation of NOA-men with maturation arrest and 56.2% in the subpopulation of NOA-males with hypospermatogenesis; these rates were significantly higher than those with the SCO (9.7 %) (10). Finally, the chance of identifying motile spermatozoa in postoperative ejaculates after varicocelectomy was significantly larger in NOA-men with hypospermatogenesis in testicular biopsy compared to NOA-men with maturation arrest or SCO. Furthermore, NOA-men with maturation arrest had a larger opportunity of sperm reappearance in postoperative semen samples than those with SCO (10).

In a very recent study (44), it was confirmed that testicular histology might be of value in identifying the NOA-males who are most likely to have a benefit concerning the appearance of spermatozoa in these men after varicocele repair. There was consistently more postoperative spermatogenesis in hypospermatogenesis patients compared with SCO patients, who had very low rates of appearance of spermatozoa in the ejaculates. In a similar fashion, Ustuner et al. (60) found a certain degree of amelioration in the histological results of certain patients after varicocele repair surgery. In patients with a high probability of post-varicocele repair complications, performing TESE or microdissection-Testicular Sperm Extraction (micro-TESE) prior to varicocelectomymay be suggested. Furthermore, there is a significant probability for azoospermia relapse.

It should be mentioned that some studies do not demonstrate a beneficial effect of varicocelectomy in NOA-men on the appearance of spermatozoa in the ejaculate post-varicocelectomy in males with SCO: in fact, Kadioglu et al. (45) and Cakan et al. (46) claim that the performance of varicocelectomy in NOA-males with pure SCO is not accompanied by the appearance of spermatozoa in the ejaculate. Testicular histopathology of the patients in the former study (45) who finally demonstrated postoperative improvement revealed maturation arrest at spermatid stage (n = 3), SCO syndrome pattern with focal spermatogenesis (n = 1), and hypospermatogenesis (n = 1). In the latter study (46), induction of spermatogenesis post-varicocele repair was achieved in 3 patients. Two of them had hypospermatogenesis pre-operatively, and one had late maturation arrest. No pregnancies were achieved by natural intercourse. All men with SCO syndrome or early maturation arrest remained azoospermic after surgery (46).

Thus, several studies have provided predictive histological testicular patterns indicating the subpopulation of NOA-males in whom the repair of palpable varicocele improves sperm production. In later men, the induction of spermatogenesis following varicocelectomy contributes to the reappearance of spermatozoa to the ejaculation offering a limited probability to the couple to achieve spontaneously (8).

In addition, some studies do not demonstrate a statistically significant positive effect of varicocelectomy on sperm retrieval rate (SRR) in NOA men (47, 48). For the latter subpopulations of azoospermic males, it may be suggested that the damage in the Sertoli cellular secretory function is irreversible, and the reduction in testicular temperature offered by varicocelectomy may not be sufficient to reverse the detrimental effects of varicocele on testicular exocrine function (9).

Many relevant studies have reported that the repair of palpable varicocele improves sperm production. In the later men, the induction of spermatogenesis following varicocelectomy contributes to the reappearance of spermatozoa to the ejaculation offering a limited probability to the couple to achieve spontaneously (8, 10).

### Genetic Tests

In NOA-males with clinical varicocele, the minimal added morbidity of a diagnostic testicular biopsy at the time of varicocelectomy may be worth in order to gain significant and important information (49). However, it is of utmost importance to discover less invasive prospective indicators predicting the beneficial effects of varicocele repair. Novel molecular or genetic parameters are needed to predict high overall (50).

Comparative evaluation of 23,003 genes between subpopulations of NOA-males (with maturation arrest) positive or negative for spermatozoa in semen samples demonstrated a certain number of genes that were up-regulated, and some genes that were down-regulated in men with sperm recovery post-varicocelectomy (50).

Proliferating cell nuclear antigen expression was significantly higher in males who responded positively to varicocelectomy than in males who demonstrated a negative response (51).

### Other Parameters

Varicocele repair in a general group of infertile males with varicoceles may restore the male reproductive potential in patients with a combined testicular volume of at least 30 mL and peripheral serum follicle stimulation hormone (FSH) profiles lower than 11.7 mIU/ml (52). No relationship was observed between varicocele repair result and pre-operative serum FSH and luteinizing hormone (LH). In addition, it has been demonstrated that testicular volume and peripheral serum FSH levels are independent predictors (52).

Research efforts have demonstrated that miR-192a in seminal plasma may have a role as a predictive factor for appears the existence of spermatozoa in the semen post-varicocele repair in NOA men with varicoceles (53). Furthermore, transcriptome investigations in males with maturation arrest disclosed a well-defined difference in certain genes between varicocele repair responded males and non-responded males.

Giannakis et al. (54) demonstrated the usefulness of the testicular tissue telomerase assay in identifying NOA-males with varicoceles who do not have testicular spermatozoa but who will become positive or negative for testicular spermatozoa (either ejaculated or testicular) after varicocelectomy.

It should be emphasized that a recent study (55) did not show any correlations between testicular volume, histopathology, hormonal treatment and varicocelectomy with favorable micro-TESE outcomes in NOA-males undergone re-TESE.

## Motile Sperm Count in the Ejaculate and Natural or Assisted Pregnancy Rates Post-Varicocelectomy

As we have above mentioned, varicocelectomy in NOA-men may be accompanied by the appearance of motile spermatozoa in the ejaculates. Sajadi et al. (56) reported that microsurgical varicocelectomy in NOA-men has a beneficial effect on sperm appearance in ejaculates postoperatively, and it may result in natural or assisted pregnancies.

There was evidence of the appearance of spermatozoa in the postoperative semen analysis in 43.9% (151/344) of patients in a systematic review and meta-analysis that included 16 studies with 344 NOA-men (10). The range of findings was 20.8% to 55.0%. The mean, standard deviation postoperative sperm density was 1.82 × 10^6^ ± 1.58 × 10^6^ ml^−1^, and the mean sperm motility was 22.9% ± 15.5% (10).

In an interesting and clinically significant meta-analysis, Weedin et al. reported the appearance of motile sperm in post-varicocele repair collected semen samples in 91 of 233 (39.1%) men. The authors emphasized the presence of 14 (6%) spontaneous pregnancies and ten pregnancies with the assistance of IVF. The mean ± SD postoperative sperm count, and motility were 1.6 × 10^6^ × 1.2 × 10^6^ and 20.1% ±18.5%, respectively. Eleven (4.6%) patients of the NOA group presenting motile sperm in the ejaculate postoperatively had relapsed into azoospermia within 2–6 months (8).

A significant percentage of NOA-men with clinically palpable varicocele has acquired biological offspring with the natural selection process avoiding assisted reproductive techniques (57). Despite these initial encouraging results, varicocele repair in NOA-men with clinical varicocele is not recommended as a routine treatment. Some studies have presented that NOA-men with clinical varicocele rarely have enough spermatozoa in their ejaculate after varicocelectomy to avoid TESE. In fact, Schlegel and Kaufmann published those seven out of 31 patients (22%) had demonstrated spermatozoa in their ejaculates after surgery, with an average follow-up of 14.7 months. Among them, a percentage equal to 9.6% only had sufficient motile spermatozoa in ejaculation to participate in the ICSI technique and avoid TESE (48).

The appearance of motile sperm in the semen analysis of NOA-men who have undergone varicocele repair may not be permanent. Over time, spermatozoa may not be detected in the semen, and subsequently, azoospermia is relapsing (5, 7). In a study by Pasqualotto et al. (58), 27 NOA-men underwent microsurgical varicocelectomy, and 6 months after the surgery, semen samples were evaluated. According to the findings of this study, nine patients had spermatozoa in their ejaculate post-varicocelectomy. However, 12 months after surgery, five of those patients (55.6%) relapsed to azoospermia. The researchers have hypothesized that the appearance of spermatozoa in the ejaculate following surgery could be a temporary effect of the surgery's induction of spermatogenesis. Consequently, some researchers suggest as mandatory the cryopreservation of motile sperm samples after the repair of varicocele in NOA-men (5, 7, 58).

**Figure 2:**
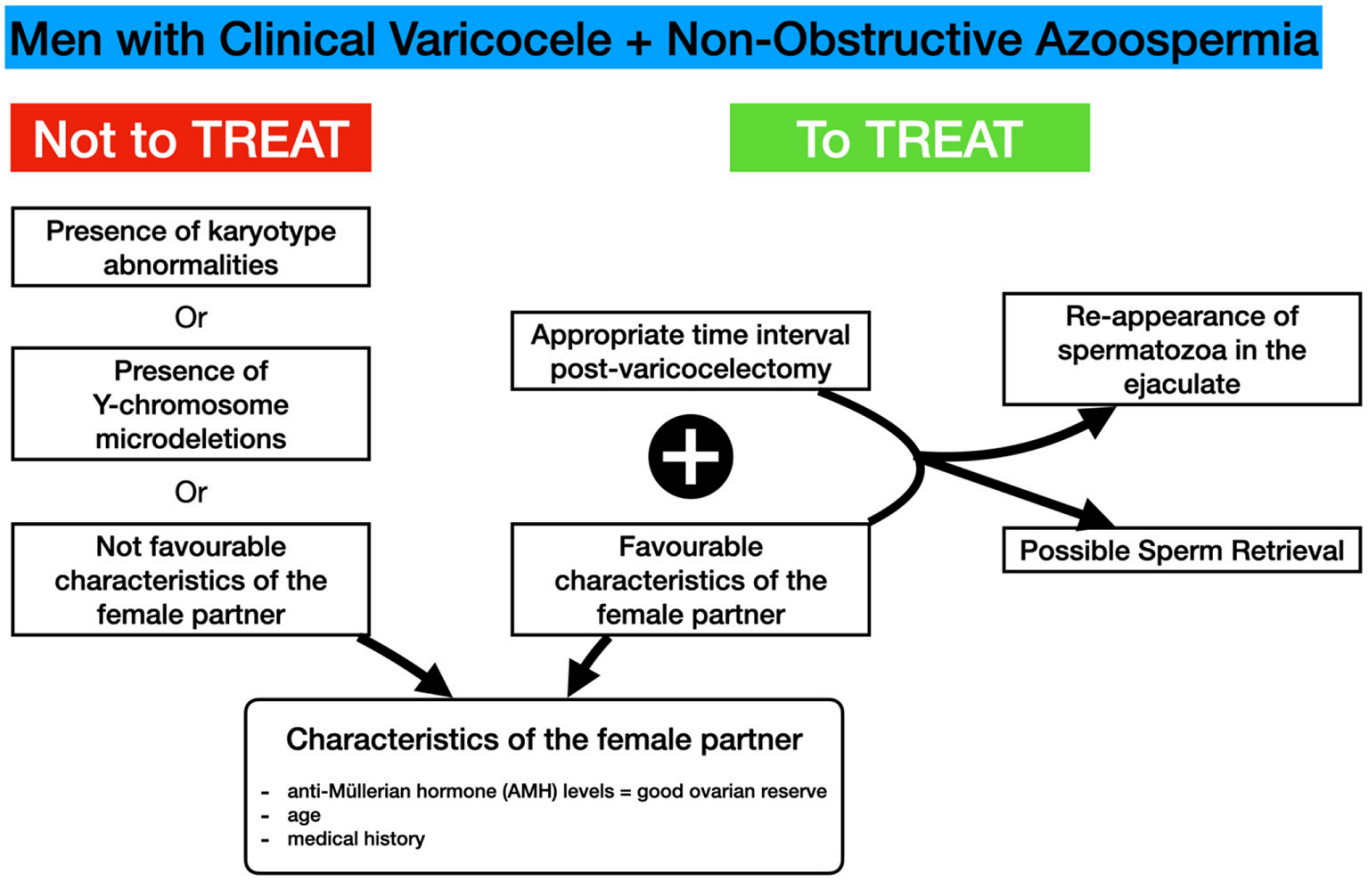
Algorithm for the therapeutic management of NOA-men with varicoceles.

Although not indicated in many studies, the period between varicocele repair and sperm reappearance in the ejaculate varies from 4.5 months to 11 months. Unfortunately, sometimes the female age does not permit waiting for the beneficial effects of varicocelectomy (49) (Figure 2).

It should be emphasized that the studies published in PubMed basis concerning the outcome of varicocelectomy in NOA-males are highly variable. Looking through Table 1, it is obvious that the outcome in the percentage of males with motile spermatozoa in the ejaculates ranges from 5% (59) to 69% (60).

**Table 1:** Studies describing the outcome of varicocelectomy on the appearance of spermatozoa in semen, SRR, and pregnancy rates post-ICSI techniques.

**References**	**No. of patients**	**Procedure approach**	**Presence of sperm in postoperative ejaculate**		**Azoospermia relapse**	**Sperm retrieval**		**Pregnancy rates**	
			**No. (%) of patients with motile sperm**	**Total no. of motile sperm (x 10^**6**^)**		**SRR**	**Histopathology**	**Natural**	**With ART**
Matthew et al. (7)	22	Microsurgical varicocelectomy	12/22 (55%)	2.2 ± 1.1	–	–	–	2/22 (9%)	1/22 (5%)
Kim et al. (96)	28	Varicocelectomy inguinal	12/28 (43%)	1.2 ± 3.6	2/12 (17%)	–	HYPO: 18, MA: 13, SCO: 3	–	2/28 (7%)
Kadioglu et al. (45)	24	Microsurgical varicocelectomy	5/24 (21%)	0.04 ± 0.03	–	–	HYPO:3, MA: 14, SCO: 7	0/24 (0%)	–
Schlegel et al. (48)	31	Microsurgical varicocelectomy	7/31 (22%)	–	4/7 (57%)	–	–	0/31 (0%)	–
Giannakiset al. (54)	22	Microsurgery subinguinal	6/22 (27%)	2	–	–	–	–	–
Cakanet al. (46)	13	Varicocelectomy inguinal	3/13 (23%)	0.7	0/13 (0%)	–	HYPO: 5, MA: 3, SCO: 5	0/13 (0%)	–
Esteves et al. (97)	17	Microsurgery inguinal	6/17 (35%)	0.8	–	4/9 (44.4%)	HYPO: 6, MA: 5, SCO: 6	1/17 (6%)	–
Gat et al. (98)	32	Percutaneous embolization	18/32 (56%)	3.8	7/18 (39%)	–	–	4/32 (13%)	5/32 (16%)
Pasqualotto et al. (58)	27	Microsurgery subinguinal	9/27 (33%)	0.87 ± 1.74	7/9 (78%)	–	HYPO: 9, MA: 8, SCO: 10	1/9 (11%)	–
Poulakis et al. (99)	14	Anterograde sclerotherapy	7/14 (50%)	3.1 ± 1.2	–	–	HYPO: 4, MA: 5, SCO: 3	2/14 (14%)	–
Lee et al. (5)	19	Microsurgery inguinal	7/19 (37%)	0.36	2/7 (29%)	–	HYPO: 3, MA: 6, SCO: 10	1/19 (5%)	–
Ishikawa et al. (100)	6	Microsurgery inguinal	2/6 (33%)	0.2	–	–	–	0/6 (0%)	–
Youssef et al. (101)	54	High ligation	14/54 (26%)	3.56 ± 4.8	–	–			–
Cocuzza et al. (102)	10	Microsurgery subinguinal	3/10 (30%)	5.5	–	–	HYPO: 2, MA: 4, SCO: 4	–	–
Inciet al. (62)	66	Microsurgical varicocelectomy	–	–	–	35/66 (53%)	–	–	11/66 (17%)
Haydardedeoglu et al. (63)	74	Macrosurgical varicocelectomy	–	–	–	45/74 (60.8%)	–	–	23/74 (31%)
Abdel-Meguid et al. (78)	31	Microsurgery subinguinal	9/31 (29%)	2.3 ± 1.7	2/9 (22%)	10/31 (32%)	HYPO: 13, MA: 6, SCO: 2	–	–
Kiracet al. (103)	23	Microsurgery subinguinal	7/23 (30%)	1.34	–	–	–	1/23 (4%)	2/23 (9%)
Zampieriet al. (64)	35	Varicocelectomy	17/35 (49%)	0.6	–	15/35 (43%)	–	0/35 (0%)	0/35 (0%)
Aboutaleb et al. (42)	20	Loupe-assisted subinguinal	6/20 (30%)	2	–	–	HYPO: 7, MA: 3, SCO: 10	–	–
D'Andrea et al. (104)	23	Embolization	11/23 (48%)	1.3	–	–	–	–	–
Ustuneret al. (59)	19	Microsurgery subinguinal	1/19 (5%)	–	–	8/19 (42%)	HYPO: 2, MA: 3, SCO: 14	–	–
Shiraishiet al. (51)	83	Microsurgery inguinal	20/83 (24%)	7.8	–	30/83 (36%)	HYPO: 13, MA: 27, SCO: 43	–	5/83 (6%)
Sajadiet al. (56)	57	Microsurgical varicocelectomy	8/57 (14%)	–	–	14/38 (37%)	HYPO: 3, MA: 8, SCO: 3	1/57 (2%)	1/57 (2%)
Elbardisiet al. (105)	42	Microsurgical varicocelectomy	11/42 (26%)	1.98 ± 5.4	0/11 (0%)	–	HYPO: 8, MA: 0, SCO: 3	–	–
Birowoet al. (60)	42	Microsurgery subinguinal	29/42 (69%)	–	–	–	HYPO: 0, MA: 42, SCO: 0	–	–

## Sperm Retrieval Rate in Males With Non-Obstructive Azoospermia After Varicocelectomy and Outcome of ICSI

Furthermore, in NOA-men who did not demonstrate spermatozoa in ejaculates post-varicocelectomy, it has been reported that varicocelectomy increases the SRR and increases the pregnancy rate post-ICSI. In addition to the effects on male reproductive potential, varicocelectomy improves the secretory function of Leydig cells, which results in an improvement of androgen production, negating the need for testosterone replacement therapy (61).

Although spermatogenesis after varicocelectomy improves in 39.1% of a population of NOA-males and may lead to the appearance of sperm in the seminal fluid, TESE is inevitable in some patients, post-varicocelectomy, due to insufficient motile sperm count in ejaculation and due to recurrence of azoospermia in other patients (8, 10, 48, 58).

There are few studies evaluating the influence of varicocelectomy on the outcome of TESE. A meta-analysis of three studies evaluating the SRR in 241 NOA-men with clinical varicocele demonstrated significantly (2.65-fold) higher SRR in NOA-men who underwent varicocelectomy compared to NOA-men with untreated varicocele (10).

Inci et al. evaluated SRR in specimens recovered by micro-TESE and the subsequent results of ICSI techniques in 96 NOA-men. This study included 66 NOA-men with clinical varicocele who had previously undergone varicocelectomy and 30 NOA-men with untreated clinical varicocele. This study has demonstrated that varicocelectomy significantly increases SRR in NOA-men with clinical palpable varicocele (53 vs. 30%) (62).

Haydardedeoglu et al. also compared outcomes of SRR and ICSI in two groups, the varicocele repair group vs. the non-repair group. They reported that SRR was higher in the group of NOA-men with varicocelectomy (60.81 and 38.46%, *p* = 0.01, respectively) (63). The clinical pregnancy rate and the live birth rate, as well, were significantly larger in the males undergone varicocelectomy (74.2 vs. 52.3% and 64.5 vs. 41.5%, respectively).

Zampieri et al. (64) also reported significantly higher SRR equal to 57.8% in men treated with varicocelectomy compared with 27% in untreated NOA-men with clinical varicocele. The optimal time for sperm retrieval after varicocele repair is under investigation. It has been recommended that an interval of 3 months or longer between varicocele repair and sperm retrieval surgical procedure is recommended (65).

In contrast, Schlegel et al. (48) and Althakafi et al. (66) have suggested that varicocele repair may not be significant associates of success in NOA-men undergoing microsurgery.

## Mechanisms of Development of Testicular Damage in Varicocelized Experimental Animal Models and Therapeutic/Protective Effects of Varicocelectomy

Development of a left varicocele model in the rat (18, 67, 68), rabbit (69), monkey (70), and dog (71) demonstrated testicular endocrine and exocrine damage. Performance of varicocelectomy in varicocelized rats (72, 73) or rabbits (74) improved testicular exocrine function.

The detrimental effect of left varicocele on testicular endocrine and exocrine function has been proven in experimental animals with left varicocele (i.e., the rat model, rabbit model, dog model, or guinea pig model) (18). Taking into consideration the results of studies in experimental animal models of left varicocele, Sofikitis et al. (18) explained why a subpopulation of males with left varicocele remain fertile. Very briefly, according to this hypothesis, the latter subpopulation of fertile males with left varicocele has a very good development of the lymphatic drainage system in the testis bilaterally; therefore, the increase in the testicular extracellular fluid pressure caused by varicocele can be compensated due to the extended development of the testicular lymphatics. The above mechanism is strongly supported by studies in the monkey model of varicocele developed by Harrison et al. (75). In fact, Harrison has demonstrated that if testicular parenchymal lymphatics can carry the extra fluid load alterations caused by varicocele, no detrimental alterations in testicular function would be observed. Harrison and co-workers have demonstrated that in monkeys with varicoceles, the lymphatics cannot remove the extra fluid load fast enough. In that case, the result will be a slight increase in testicular tissue fluid pressure. Subsequently, the movement of fluid and nutrients from the circulation into the tissue beds will decrease, and the testicular tissue may become more ischemic, and cellular damage may occur. However, if there is an extensive development of lymphatics in the testicular tissue, the extracellular testicular fluid pressure will decrease with an overall result improvement in testicular function (18, 75). Furthermore, fertile males with left varicoceles may become at a later age (when they do not desire children) infertile (because of the pathophysiology of progressive testicular damage) without having understood it.

## Guidelines and Isolated Series of Studies Recommending or Rejecting the Need for Varicocelectomy in NOA-Men With Clinical Varicoceles

### Guidelines by the European Association of Urology

In NOA-men who are treated with varicocele repair, SRRs are higher than those without varicocelectomy (76). In 43.9% of the patients, spermatozoa have been found in semen samples after varicocele repair. These results suggest that varicocele repair in NOA men with clinical varicocele is associated with higher SRR. In addition, 44% of the NOA-males demonstrate the appearance of spermatozoa in the ejaculate. Thus, the latter males may avoid surgical sperm recovery (76). On the other hand, the quality of evidence available is low. Appropriate information of the NOA-man should be given concerning the risks and benefits of varicocele repair prior to the performance of surgery (76).

It should be emphasized that the European Association of Urology (EAU) Guidelines recommend TESE procedures for the therapeutic management of NOA-men (76). EAU Guidelines suggest if a diagnostic testicular biopsy is desired, the testicular biopsy must be combined with TESE for sperm cryopreservation. However, in the case of the presence of factor that may play a causative role in the development of NOA (i.e., left varicocele), the EAU Guidelines do not take a position against the therapeutic effort to retract such a causative/contributing factor. Adopting this approach, the overall objective may be the appearance of spermatozoa in the ejaculate after removal of the factor that contributes to/causes NOA (i.e., the performance of varicocelectomy in NOA-males with varicocele or the administration of GnRH/gonadotropins in individuals with hypogonadotropic hypogonadism) (76). This therapeutic approach is absolutely consistent with the principle of The Hippokratical Medicine to remove the cause of pathophysiology (i.e., varicocele) in order to treat the consequences (i.e., azoospermia) of that pathophysiology.

### Guidelines by the American Urological Association and American Society for Reproductive Medicine

There are few studies, including a small number of men with NOA that have elucidated the influence of varicocele repair on the appearance of sperm in the ejaculate post-operatively. There are no high-quality data to recommend varicocelectomy in NOA males. In addition, varicocele repair delays ART employment for at least 6 months. When varicocelectomy is suggested prior to employment of TESE combined with ART, couples should be adequately informed of the limited evidence recommending this approach (77).

### Difficulties to Design RCTs in Reproductive Medicine

It should be mentioned that almost all of the studies that evaluate the effects of varicocelectomy in NOA-men are non-randomized controlled studies or non-controlled studies. Definitely, this issue has been taken into serious consideration in the generation of Guidelines on Varicocele Repair by Scientific Societies. However, the scientific community is aware of the difficulties to perform randomized studies comparing one surgical procedure with another therapeutic mode. Definitely, it is very difficult and probably non-ethical to design a randomized study in which one arm will include a surgical therapeutic procedure, whereas the other arm of the study will not receive any treatment/or will not receive the most appropriate treatment. Patients may claim that the physicians are non-ethical violating Hippocrates Oath if they deprive a new promising treatment from a subpopulation of patients. On the other hand, looking through the international literature only one randomized controlled study was noted on the importance of performing varicocelectomy (78). The latter study demonstrated a significant and positive effect of varicocele in oligoasthenoteratospermic males. However, in that study NOA- males were not included. In subpopulations of azoospermicmales, varicocele may be a contributing factor to the development of the azoospermic phenotype. Thus, in a subpopulation of azoospermic males who demonstrated the appearance of spermatozoa in the ejaculate post-varicocele repair, the probability that another harmful contributing factor had been additionally removed/neutralized cannot be ruled out. In fact, several studies indicate the importance of discovering additional pathophysiologies to the varicocele pathophysiology that contribute to a certain degree to the phenotype of progressive testicular damage in NOA-males with varicoceles (i.e., among other contributing factors, the literature discusses the importance of smoking and obesity) (79, 80).

It should be emphasized that, in some studies dealing with the effect of varicocelectomy in NOA-men, the methodology followed for the differential diagnosis between non-obstructive azoospermia and obstructive azoospermia has not been described adequately. However, in several studies, it is clearly mentioned that varicocele was diagnosed both by clinical examination and ultrasonographical evaluation. In addition, in several studies the pellet of more than one semen samples post-centrifugation was evaluated, and the presence of genetic anomalies was investigated prior to the performance of varicocelectomy (48, 56, 81).

## Surgical Repair of Varicocele

Conventional open non-magnified varicocelectomy, laparoscopic/robotic varicocelectomy, and microsurgical varicocelectomy are varicocele's most common surgical treatment methods. There are no long-term results concerning pregnancy rates. Differences in the types and frequency of complications are the main reasons for choosing between subinguinal and supra-inguinal surgical techniques. According to the literature, failure or recurrence rates for varicocele surgery vary from 1.05% for magnified repair approach to 14.97% for high inguinal canal approaches (82).

### Subinguinal/Inguinal Microsurgical Varicocelectomy

The current gold standard therapy for varicoceles is the subinguinal microsurgical varicocelectomy. The subinguinal microscopic method indeed has several benefits, including (a) saving of the artery and the lymphatics, (b) a high success rate in terms of improving some of the standard parameters of semen analysis, and (c) a minimal risk of hydrocele.

The following arguments represent the four most significant advantages of microsurgical subinguinal varicocelectomy (in contrast to inguinal or retroperitoneal varicocelectomy): (1) a subinguinal incision offers the opportunity to expose the spermatic cord without dividing any abdominal muscles or fascia; the overall result is the development of a smaller degree of post-operative pain and a more rapid return to the normal activities of a given patient, (2) clear identification of all of the dilated veins offers the opportunity to reduce the possibility for varicocele recurrence, (3) accurate identification of the internal, external and vassal arteries removes the risk for ligation of the testicular arteries, and (4) testicular veins can be clearly identified (83).

The venous drainage of the testis additionally to the pampiniform plexus-internal spermatic vein system, includes the cremasteric veins, the periarterial venous plexus, extra-spermatic collaterals, and gubernacular collaterals. If these minor venous collaterals are not identified/ligated, their diameter will increase post-varicocele; the overall result may be varicocele recurrence having subsequently harmful effects on testicular endocrine and exocrine function. Hopps and colleagues (84) suggest blockage of any veins larger than 2.0 mm in diameter, including the gubernacular veins, which may be identified by delivering the testis above the incision and exposing the gubernaculum. It should be emphasized that the existence of significant structural polymorphisms in the veno-spermatic area (7% of cases) makes radiological therapy contraindicated, with conventional surgical procedures being chosen in these instances.

### Retroperitoneal Approach (Palomo)

The Palomo surgical procedure was historically first employed. It involves retroperitoneal ligation of the left internal spermatic vein without magnification. Disadvantages of this surgical procedure involve (a) the inability to ligate the external spermatic vein system, (b) the inability to secure the integrity of testicular arteries, and (c) the inability to avoid damaging the testicular lymphatic venous drainage system. Another major disadvantage of the Palomo technique is that it may not be possible to ligate all the left internal spermatic veins. A large number of internal spermatic veins has been identified occasionally in the lumbar level (85).

### Laparoscopic/Open Suprainguinal Varicocelectomy

During an open or laparoscopic operation, the suprainguinal approach with spermatic vein ligation can be performed easily and with a subsequent high success rate. However, even with the increased magnification provided by laparoscopy, it may be difficult to identify and preserve lymphatics and arteries.

### Sclerotherapy/Embolotherapy

Sclerosing agents, tissue adhesives, or detachable metallic coils may be used to selectively catheterize and embolize the gonadal veins as an alternative to surgical varicocele repair. Antegrade or retrograde injection of sclerosant or embolizing substance injections have been utilized for many years as treatments alternative to surgery.

Compared to open or laparoscopic surgery, retrograde and antegrade techniques have a lower success rate and need radiation exposure (86–92). An intravascular coil or balloon with or without 3% sodium tetradecyl sulfate or polidocanol may be used retrogradely to occlude the vein. It has been stated that the procedure is technically impossible in 5–22% of cases and that rates of persistence or recurrence range from 6 to 35%. Pain, epididymo-orchitis, phlebitis, and scrotal oedema are all self-limiting consequences; testicular atrophy or hydrocele are not usually observed. Antegrade techniques with or without fluoroscopic vein identification procedure involve the isolation of a vein or veins that are susceptible to cannulation, followed by injecting a sclerosant to ligate the vein (93–95). The success rate is 4 to 12% lower than the respective rate gained by retrograde sclerotherapy, but the consequences are the same.

## Conclusions

Varicocelectomy in NOA-men has a beneficial effect on spermatogenesis and the reappearance of motile spermatozoa in the ejaculate. In addition, it increases SRR in men who remain azoospermic post-varicocelectomy. However, there is a possibility that NOA-men who are positive for spermatozoa in the semen samples post-varicocele repair will relapse into azoospermia. As a result, NOA-men should be advised to freeze spermatozoa appearing the semen post-varicocelectomy. In addition, performance of varicocelectomy in NOA-men and subsequent ICSI procedures using testicular spermatozoa may increase pregnancy and live birth rates in couples without female infertility factors (10, 11).

Testicular histopathology is a reliable predictor of the success of varicocelectomy in NOA-men. Consequently, NOA-men with hypospermatogenesis in the testicular biopsy are more likely to develop motile spermatozoa on postoperative ejaculate after varicocelectomy than NOA-men with early maturation arrest and SCO. In addition, NOA-men with early or late maturation arrest are more likely to develop motile spermatozoa in postoperative ejaculates compared with SCO (10, 11).

It should be emphasized that the level of evidence for the above beneficial effects of varicocelectomy in NOA-men is low, and the beneficial or detrimental effects of varicocelectomy should be discussed with these men (10, 11).

## Author Contributions

AK, EM, AZ, and FD contributed to conception and design of the manuscript. AK wrote the first draft of the manuscript. SA, CM, IG, and PT wrote sections of the manuscript. All authors contributed to manuscript revision, read, and approved the submitted version.

## Conflict of Interest

The authors declare that the research was conducted in the absence of any commercial or financial relationships that could be construed as a potential conflict of interest.

## Publisher's Note

All claims expressed in this article are solely those of the authors and do not necessarily represent those of their affiliated organizations, or those of the publisher, the editors and the reviewers. Any product that may be evaluated in this article, or claim that may be made by its manufacturer, is not guaranteed or endorsed by the publisher.

## Abbreviations

ART: assisted reproductive technology
ASRM: American Society for Reproductive Medicine
AUA: American Urological Association
EAU: European Association of Urology
FSH: follicle stimulation hormone
ICSI: intracytoplasmic sperm injection
LH: luteinizing hormone
Micro-TESE: microdissection-Testicular Sperm Extraction
NOA: non-obstructive azoospermia
SCO: Sertoli cell-only
SRR: sperm retrieval rates
TESE: testicular sperm extraction.

## References

[1] Palermo G Joris H Devroey P Van Steirteghem AC. Pregnancies after intracytoplasmic injection of single spermatozoon into an oocyte. Lancet. (1992) 340:17–8. 10.1016/0140-6736(92)92425-f 1351601

[2] Jarow JP Espeland MA Lipshultz LI. Evaluation of the azoospermic patient. J Urol. (1989) 142:62–5. 10.1016/s0022-5347(17)38662-7 2499695

[3] World Health Organization. WHO laboratory manual for the examination and processing of human semen. 5th edition. Geneva: World Health Organization (2010). xiv, 271

[4] Schlegel PN. Causes of azoospermia and their management. Reprod Fertil Dev. (2004) 16:561–72. 10.10371/RD03087 15367371

[5] Lee JS Park HJ Seo JT. What is the indication of varicocelectomy in men with nonobstructive azoospermia? Urology. (2007) 69:352–5. 10.1016/j.urology.2006.10.010 17320677

[6] Tulloch WS. A consideration of sterility factors in the light of subsequent pregnancies. II Sub fertility in the male (Tr Edinburgh Obst Soc Session 104). Edinb Med J. (1951) 59:29–34. 24541934 PMC5274806

[7] Matthews GJ Matthews ED Goldstein M. Induction of spermatogenesis and achievement of pregnancy after microsurgical varicocelectomy in men with azoospermia and severe oligoasthenospermia. Fertil Steril. (1998) 70:71–5. 10.1016/s0015-0282(98)00108-3 9660424

[8] Weedin JW Khera M Lipshultz LI. Varicocele repair in patients with nonobstructive azoospermia: a meta-analysis. J Urol. (2010) 183:2309–15. 10.1016/j.juro.2010.02.012 20400156

[9] Agarwal A Sharma R Harlev A Esteves SC. Effect of varicocele on semen characteristics according to the new 2010 World Health Organization criteria: a systematic review and meta-analysis. Asian J Androl. (2016) 18:163–70. 10.4103/1008-682X.172638 26780872 PMC4770480

[10] Esteves SC Miyaoka R Roque M Agarwal A. Outcome of varicocele repair in men with nonobstructive azoospermia: systematic review and meta-analysis. Asian J Androl. (2016) 18:246–53. 10.4103/1008-682X.169562 26680033 PMC4770494

[11] Birowo P Tendi W Widyahening IS Atmoko W Rasyid N. The benefits of varicocele repair for achieving pregnancy in male infertility: a systematic review and meta-analysis. Heliyon. (2020) 6:e05439. 10.1016/j.heliyon.2020.e05439 33204888 PMC7648199

[12] Goldstein M Eid JF. Elevation of intratesticular and scrotal skin surface temperature in men with varicocele. J Urol. (1989) 142:743–5. 10.1016/s0022-5347(17)38874-2 2769853

[13] Su LM Goldstein M Schlegel PN. The effect of varicocelectomy on serum testosterone levels in infertile men with varicoceles. J Urol. (1995) 154:1752–5. 7563339

[14] Hudson RW McKay DE. The gonadotropin response of men with varicoceles to gonadotropin-releasing hormone. Fertil Steril. (1980) 33:427–32. 6767632

[15] Hudson RW Hayes KA Crawford VA McKay DE. Seminal plasma testosterone and dihydrotestosterone levels in men with varicoceles. Int J Androl. (1983) 6:135–42. 10.1111/j.1365-2605.1983.tb00332.x 6862670

[16] Cayan S Akbay E Saylam B Kadioglu A. Effect of varicocele and its treatment on testosterone in hypogonadal men with varicocele: review of the literature. Balkan Med J. (2020) 37:121–4. 10.4274/balkanmedj.galenos.2020.2020.1.85 32070086 PMC7161614

[17] Ando S Giacchetto C Colpi G Beraldi E Panno ML Lombardi A. Physiopathologic aspects of Leydig cell function in varicocele patients. J Androl. (1984) 5:163–70. 10.1002/j.1939-4640.1984.tb02388.x 6430851

[18] Sofikitis N Stavrou S Skouros S Dimitriadis F Tsounapi P Takenaka A. mysteries, facts, and fiction in varicocele pathophysiology and treatment. Eur Urol Suppl. (2014) 13:89–99. 10.1016/j.eursup.2014.07.002

[19] Comhaire F Vermeulen A. Varicocele sterility: cortisol and catecholamines. Fertil Steril. (1974) 25:88–95. 10.1016/s0015-0282(16)40159-7 4810205

[20] Russell LD Brinster RL. Ultrastructural observations of spermatogenesis following transplantation of rat testis cells into mouse seminiferous tubules. J Androl. (1996) 17:615–27. 9016391

[21] Sofikitis N Ono K Yamamoto Y Papadopoulos H Miyagawa I. Influence of the male reproductive tract on the reproductive potential of round spermatids abnormally released from the seminiferous epithelium. Hum Reprod. (1999) 14:1998–2006. 10.1093/humrep/14.8.1998 10438417

[22] Cozzolino DJ Lipshultz LI. Varicocele as a progressive lesion: positive effect of varicocele repair. Hum Reprod Update. (2001) 7:55–8. 10.1093/humupd/7.1.55 11212075

[23] Dimitriadis F Tsiampali C Chaliasos N Tsounapi P Takenaka A Sofikitis N. The Sertoli cell as the orchestra conductor of spermatogenesis: spermatogenic cells dance to the tune of testosterone. Hormones (Athens). (2015) 14:479–503. 10.14310/horm.2002.1633 26732153

[24] Goulis D Mintziori G Koliakos N Hatzichristou D Papadimas I Hatzimouratidis K. Inhibin B and anti-Mullerian hormone in spermatic vein of subfertile men with varicocele. Reprod Sci. (2011) 18:551–5. 10.1177/1933719110393024 21285453

[25] Namiki M Nakamura M Okuyama A Sonoda T Nishimune Y Takatsuka D. Influence of temperature on the function of Sertoli and Leydig cells of human testes. Fertil Steril. (1987) 47:475–80. 10.1016/s0015-0282(16)59058-x 3556625

[26] Kavoussi PK Hunn C Gilkey MS Chen SH Kavoussi KM Wininger JD. Sertoli cell only syndrome induced by a varicocele. Transl Androl Urol. (2019) 8:405–8. 10.21037/tau.2019.06.17 31555565 PMC6732098

[27] Gat Y Zukerman Z Chakraborty J Gornish M. Varicocele, hypoxia and male infertility. Fluid Mechanics analysis of the impaired testicular venous drainage system. Hum Reprod. (2005) 20:2614–9. 10.1093/humrep/dei089 15932914

[28] Wright EJ Young GP Goldstein M. Reduction in testicular temperature after varicocelectomy in infertile men. Urology. (1997) 50:257–9. 10.1016/s0090-4295(97)00191-x 9255298

[29] Zorgniotti AW Sealfon AI Toth A. Chronic scrotal hypothermia as a treatment for poor semen quality. Lancet. (1980) 1:904–6. 10.1016/s0140-6736(80)90839-9 6103260

[30] Zorgniotti AW Sealfon AI. Scrotal hypothermia: new therapy for poor semen. Urology. (1984) 23:439–41. 10.1016/s0090-4295(84)80006-0 6372197

[31] Abdel-Meguid TA Farsi HM Al-Sayyad A Tayib A Mosli HA Halawani AH. Effects of varicocele on serum testosterone and changes of testosterone after varicocelectomy: a prospective controlled study. Urology. (2014) 84:1081–7. 10.1016/j.urology.2014.05.029 25214202

[32] Chen X Yang D Lin G Bao J Wang J Tan W. Efficacy of varicocelectomy in the treatment of hypogonadism in subfertile males with clinical varicocele: a meta-analysis. Andrologia. (2017) 49:12778. 10.1111/and.12778 28378913

[33] Alizadeh M Nasebakht A Valizadeh R Mohammadi Fallah M Taghizadeh Afshari A Rahimi MM. A preliminary evaluation of serum level of testosterone, LH, and FSH in patients with varicocele after varicocelectomy as a kidney-related disease. Ther Clin Risk Manag. (2018) 14:1585–90. 10.2147/TCRM.S161641 30233193 PMC6129023

[34] Sathya Srini V Belur Veerachari S. Does varicocelectomy improve gonadal function in men with hypogonadism and infertility? Analysis of a prospective study. Int J Endocrinol. (2011) 2011:916380. 10.1155/2011/916380 22190930 PMC3235454

[35] Wan X Wang H Ji Z. Microsurgical varicocelectomy for clinical varicocele: a review for potential new indications. Andrologia. (2017) 49:12827. 10.1111/and.12827 28671268

[36] Schlegel PN Goldstein M. Alternate indications for varicocele repair: non-obstructive azoospermia, pain, androgen deficiency and progressive testicular dysfunction. Fertil Steril. (2011) 96:1288–93. 10.1016/j.fertnstert.2011.10.033 22130099

[37] Pierik FH Abdesselam SA Vreeburg JT Dohle GR De Jong FH Weber RF. Increased serum inhibin B levels after varicocele treatment. Clin Endocrinol (Oxf). (2001) 54:775–80. 10.1046/j.1365-2265.2001.01302.x 11422112

[38] Moubasher AE Taha EA Younis A Fakhry ME Morsy H. Testicular tissue oxidative stress in azoospermic patients: effect of cryopreservation. Andrologia. (2020) 52:e13817. 10.1111/and.13817 32920894

[39] Mostafa T Anis TH El-Nashar A Imam H Othman IA. Varicocelectomy reduces reactive oxygen species levels and increases antioxidant activity of seminal plasma from infertile men with varicocele. Int J Androl. (2001) 24:261–5. 10.1046/j.1365-2605.2001.00296.x 11554982

[40] Agarwal A Sharma RK Desai NR Prabakaran S Tavares A Sabanegh E. Role of oxidative stress in pathogenesis of varicocele and infertility. Urology. (2009) 73:461–9. 10.1016/j.urology.2008.07.053 19167039

[41] Abarikwu SO Pant AB Farombi EO. Dietary antioxidant, quercetin, protects sertoli-germ cell coculture from atrazine-induced oxidative damage. J Biochem Mol Toxicol. (2012) 26:477–85. 10.1002/jbt.21449 23132811

[42] Aboutaleb HA Elsherif EA Omar MK Abdelbaky TM. Testicular biopsy histopathology as an indicator of successful restoration of spermatogenesis after varicocelectomy in non-obstructive azoospermia. World J Mens Health. (2014) 32:43–9. 10.5534/wjmh.2014.32.1.43 24872951 PMC4026233

[43] Majzoub A ElBardisi H Covarrubias S Mak N Agarwal A Henkel R. Effect of microsurgical varicocelectomy on fertility outcome and treatment plans of patients with severe oligozoospermia: An original report and meta-analysis. Andrologia. (2021) 53:e14059. 10.1111/and.14059 33763931

[44] Jensen S Ko EY. Varicocele treatment in non-obstructive azoospermia: a systematic review. Arab J Urol. (2021) 19:221–6. 10.1080/2090598X.2021.1956838 34552773 PMC8451638

[45] Kadioglu A Tefekli A Cayan S Kandirali E Erdemir F Tellaloglu S. Microsurgical inguinal varicocele repair in azoospermic men. Urology. (2001) 57:328–33. 10.1016/s0090-4295(00)00908-0 11182347

[46] Cakan M Altug U. Induction of spermatogenesis by inguinal varicocele repair in azoospermic men. Arch Androl. (2004) 50:145–50. 10.1080/01485010490425250 15204679

[47] Kizilkan Y Toksoz S Turunc T Ozkardes H. Parameters predicting sperm retrieval rates during microscopic testicular sperm extraction in nonobstructive azoospermia. Andrologia. (2019) 51:e13441. 10.1111/and.13441 31583760

[48] Schlegel PN Kaufmann J. Role of varicocelectomy in men with nonobstructive azoospermia. Fertil Steril. (2004) 81:1585–8. 10.1016/j.fertnstert.2003.10.036 15193481

[49] Najari BB. The role of varicocelectomy and diagnostic testis biopsy in men with non-obstructive azoospermia: NYU case of the month, July 2020. Rev Urol. (2020) 22:130–2. 33239973 PMC7672499

[50] Abdel-Meguid TA. Can we reliably predict sperm recovery in semen of nonobstructive azoospermia men after varicocele repair?-answers are awaited. Transl Androl Urol. (2017) 6:317–9. 10.21037/tau.2017.02.02 28540245 PMC5422689

[51] Shiraishi K Oka S Matsuyama H. Predictive factors for sperm recovery after varicocelectomy in men with nonobstructive azoospermia. J Urol. (2017) 197:485–90. 10.1016/j.juro.2016.08.085 27545577

[52] Yoshida K Kitahara S Chiba K Horiuchi S Horimi H Sumi S. Predictive indicators of successful varicocele repair in men with infertility. Int J Fertil Womens Med. (2000) 45:279–84. 10997484

[53] Zhi EL Liang GQ Li P Chen HX Tian RH Xu P. Seminal plasma miR-192a: a biomarker predicting successful resolution of nonobstructive azoospermia following varicocele repair. Asian J Androl. (2018) 20:396–9. 10.4103/aja.aja_8_18 29578116 PMC6038165

[54] Giannakis D Baltogiannis D Tsoukanelis K Loutradis D Miyagawa I Makrydimas G. Role of testicular tissue telomerase assay for the prediction of the presence of testicular spermatozoa in azoospermic men with varicoceles, pre- and post-varicocelectomy. Andrologia. (2004) 36:111–22. 10.1111/j.1439-0272.2004.00615.x 15206910

[55] Amer MK Ahmed AR Hamid AAA GamalEl Din SF. Factors determining the sperm retrieval rate in fresh versus salvage micro-TESE: a comparative cohort study. Int Urol Nephrol. (2019) 51:401–8. 10.1007/s11255-019-02086-5 30701399

[56] Sajadi H Hosseini J Farrahi F Dadkhah F Sepidarkish M Sabbaghian M. Varicocelectomy may improve results for sperm retrieval and pregnancy rate in non-obstructive azoospermic men. Int J Fertil Steril. (2019) 12:303–5. 10.22074/ijfs.2019.5344 30291690 PMC6186284

[57] Inci K Gunay LM. The role of varicocele treatment in the management of non-obstructive azoospermia. Clinics. (2013) 68 Suppl 1:89–98. 10.6061/clinics/2013(sup01)10 23503958 PMC3583153

[58] Pasqualotto FF Sobreiro BP Hallak J Pasqualotto EB Lucon AM. Induction of spermatogenesis in azoospermic men after varicocelectomy repair: an update. Fertil Steril. (2006) 85:635–9. 10.1016/j.fertnstert.2005.08.043 16500331

[59] Ustuner M Yilmaz H Yavuz U Ciftci S Saribacak A Aynur BS. Varicocele repair improves testicular histology in men with nonobstructive azoospermia. Biomed Res Int. (2015) 2015:709452. 10.1155/2015/709452 26601110 PMC4639637

[60] Birowo P Prasetyo DT Pujianto DA Atmoko W Rasyid N Sini IR. Effect of varicocele repair on sperm retrieval rate and testicular histopathological patterns in men with nonobstructive azoospermia. Asian J Androl. (2022) 24:85–9. 10.4103/aja.aja_29_21 34003172 PMC8788600

[61] Bernie HL Goldstein M. Varicocele repair versus testosterone therapy for older hypogonadal men with clinical varicocele and low testosterone. Eur Urol Focus. (2018) 4:314–6. 10.1016/j.euf.2018.09.017 30316825

[62] Inci K Hascicek M Kara O Dikmen AV Gurgan T Ergen A. Sperm retrieval and intracytoplasmic sperm injection in men with nonobstructive azoospermia, and treated and untreated varicocele. J Urol. (2009) 182:1500–5. 10.1016/j.juro.2009.06.028 19683732

[63] Haydardedeoglu B Turunc T Kilicdag EB Gul U Bagis T. The effect of prior varicocelectomy in patients with nonobstructive azoospermia on intracytoplasmic sperm injection outcomes: a retrospective pilot study. Urology. (2010) 75:83–6. 10.1016/j.urology.2009.09.023 19913887

[64] Zampieri N Bosaro L Costantini C Zaffagnini S Zampieri G. Relationship between testicular sperm extraction and varicocelectomy in patients with varicocele and nonobstructive azoospermia. Urology. (2013) 82:74–7. 10.1016/j.urology.2013.03.037 23680120

[65] Practice Committee of the American Society for Reproductive Medicine. Electronic address aao. Management of nonobstructive azoospermia: a committee opinion. Fertil Steril. (2018) 110:1239–45. 10.1016/j.fertnstert.2018.09.012 30503112

[66] Althakafi SA Mustafa OM Seyam RM Al-Hathal N Kattan S. Serum testosterone levels and other determinants of sperm retrieval in microdissection testicular sperm extraction. Transl Androl Urol. (2017) 6:282–7. 10.21037/tau.2017.02.04 28540237 PMC5422690

[67] Pan J Zhu Z Xu G Niu L Yu L Luo Z. Expression of claudin11 in a rat model of varicocele and its effects on the bloodtestis barrier. Mol Med Rep. (2018) 18:5647–51. 10.3892/mmr.2018.9603 30365105 PMC6236223

[68] Hurt GS Howards SS Turner TT. Repair of experimental varicoceles in the rat. Long-term effects on testicular blood flow and temperature and cauda epididymidal sperm concentration and motility. J Androl. (1986) 7:271–6. 10.1002/j.1939-4640.1986.tb00928.x 3771366

[69] Snydle FE Cameron DF. Surgical induction of varicocele in the rabbit. J Urol. (1983) 130:1005–9. 10.1016/s0022-5347(17)51617-1 6632087

[70] Harrison RM Lewis RW Roberts JA. Pathophysiology of varicocele in nonhuman primates: long-term seminal and testicular changes. Fertil Steril. (1986) 46:500–10. 3743802

[71] Al-Juburi A Pranikoff K Dougherty KA Urry RL Cockett AT. Alteration of semen quality in dogs after creation of varicocele. Urology. (1979) 13:535–9. 10.1016/0090-4295(79)90466-7 442380

[72] Sofikitis N Takahashi C Kadowaki H Okazaki T Nakamura I Shimamoto T. Surgical repair versus medical treatment of varicocele in the rat: pharmacological manipulation of the varicocelized testicle. Eur Urol. (1992) 22:44–52. 10.1159/000474720 1425845

[73] Sofikitis N Takahashi C Nakamura I Hirakawa S Miyagawa I. Surgical repair of secondary right varicocele in rats with primary left varicocele: effects on fertility, testicular temperature, spermatogenesis, and sperm maturation. Arch Androl. (1992) 28:43–52. 10.3109/01485019208987679 1550427

[74] Sofikitis N Miyagawa I. Bilateral effect of unilateral varicocele on testicular metabolism in the rabbit. Int J Fertil Menopausal Stud. (1994) 39:239–47. 7951408

[75] Harrison RM Lewis RW Roberts JA. Testicular blood flow and fluid dynamics in monkeys with surgically induced varicoceles. J Androl. (1983) 4:256–60. 10.1002/j.1939-4640.1983.tb02363.x 6618996

[76] Salonia CB Carvalho J Corona G Jones TH Kadioglu A Martinez-Salamanca JI. Verze members of the EAU Sexual Reproductive Health Guidelines Panel. EAU Guidelines on Sexual Reproductive Health (2021). Available online at:https://uroweb.org/guideline/sexual-and-reproductive-health/

[77] Schlegel PN Sigman M Collura B De Jonge CJ Eisenberg ML Lamb DJ. Diagnosis and treatment of infertility in men: AUA/ASRM guideline PART II. J Urol. (2021) 205:44–51. 10.1097/JU.0000000000001520 33295258

[78] Abdel-Meguid TA Al-Sayyad A Tayib A Farsi HM. Does varicocele repair improve male infertility? An evidence-based perspective from a randomized, controlled trial. Eur Urol. (2011) 59:455–61. 10.1016/j.eururo.2010.12.008 21196073

[79] Antoniassi MP Belardin LB Camargo M Intasqui P Carvalho VM Cardozo KHM. Seminal plasma protein networks and enriched functions in varicocele: effect of smoking. Andrologia. (2020) 52:e13562. 10.1111/and.13562 32150769

[80] El-Dighidy MA Sherief MH Shamaa MA El-Sakka AI. Smoking and obesity negatively affect the favourable outcome of varicocelectomy in sub-fertile men. Andrologia. (2021) 53:e14131. 10.1111/and.14131 34117798

[81] Lee HC Jeong YM Lee SH Cha KY Song SH Kim NK. Association study of four polymorphisms in three folate-related enzyme genes with non-obstructive male infertility. Hum Reprod. (2006) 21:3162–70. 10.1093/humrep/del280 16861746

[82] Cayan S Shavakhabov S Kadioglu A. Treatment of palpable varicocele in infertile men: a meta-analysis to define the best technique. J Androl. (2009) 30:33–40. 10.2164/jandrol.108.005967 18772487

[83] Chan P. Management options of varicoceles. Indian J Urol. (2011) 27:65–73. 10.4103/0970-1591.78431 21716892 PMC3114590

[84] Hopps CV Lemer ML Schlegel PN Goldstein M. Intraoperative varicocele anatomy: a microscopic study of the inguinal versus subinguinal approach. J Urol. (2003) 170:2366–70. 10.1097/01.ju.0000097400.67715.f8 14634418

[85] Sofikitis N Dritsas K Miyagawa I Koutselinis A. Anatomical characteristics of the left testicular venous system in man. Arch Androl. (1993) 30:79–85. 10.3109/01485019308987738 8470944

[86] Reyes BL Trerotola SO Venbrux AC Savader SJ Lund GB Peppas DS. Percutaneous embolotherapy of adolescent varicocele: results and long-term follow-up. J Vasc Interv Radiol. (1994) 5:131–4. 10.1016/s1051-0443(94)71469-x 8136590

[87] Mazzoni G Fiocca G Minucci S Pieri S Paolicelli D Morucci M. Varicocele: a multidisciplinary approach in children and adolescents. J Urol. (1999) 162:1755–7; discussion 7-8. 10.1016/s0022-5347(05)68232-8 10524931

[88] Alqahtani A Yazbeck S Dubois J Garel L. Percutaneous embolization of varicocele in children: a Canadian experience. J Pediatr Surg. (2002) 37:783–5. 10.1053/jpsu.2002.32287 11987101

[89] Sivanathan C Abernethy LJ. Retrograde embolisation of varicocele in the paediatric age group: a review of 10 years' practice. Ann R Coll Surg Engl. (2003) 85:50–1. 10.1308/003588403321001453 12585634 PMC1964351

[90] Beutner S May M Hoschke B Helke C Lein M Roigas J. Treatment of varicocele with reference to age: a retrospective comparison of three minimally invasive procedures. Surg Endosc. (2007) 21:61–5. 10.1007/s00464-005-0684-6 17024538

[91] Granata C Oddone M Toma P Mattioli G. Retrograde percutaneous sclerotherapy of left idiopathic varicocele in children: results and follow-up. Pediatr Surg Int. (2008) 24:583–7. 10.1007/s00383-008-2124-x 18365215

[92] Reiner E Pollak JS Henderson KJ Weiss RM White RI Jr. Initial experience with 3% sodium tetradecyl sulfate foam and fibered coils for management of adolescent varicocele. J Vasc Interv Radiol. (2008) 19:207–10. 10.1016/j.jvir.2007.08.013 18341950

[93] Ficarra V Sarti A Novara G Dalpiaz O Galfano A Cavalleri S. Modified antegrade scrotal sclerotherapy in adolescent patients with varicocele. J Pediatr Surg. (2004) 39:1034–6. 10.1016/j.jpedsurg.2004.03.059 15213893

[94] Zaupa P Mayr J Hollwarth ME. Antegrade scrotal sclerotherapy for treating primary varicocele in children. BJU Int. (2006) 97:809–12. 10.1111/j.1464-410X.2006.06033.x 16536779

[95] Carmignani L Casellato S Galasso G Bozzini G Spinelli M. Dell'Agnola CA. Sclerotherapy of the pampiniform plexus with modified Marmar technique in children and adolescents. Urol Int. (2009) 82:187–90. 10.1159/000200798 19322008

[96] Kim ED Leibman BB Grinblat DM Lipshultz LI. Varicocele repair improves semen parameters in azoospermic men with spermatogenic failure. J Urol. (1999) 162:737–40. 10.1097/00005392-199909010-00031 10458356

[97] Esteves SC Glina S. Recovery of spermatogenesis after microsurgical subinguinal varicocele repair in azoospermic men based on testicular histology. Int Braz J Urol. (2005) 31:541–8. 10.1590/s1677-55382005000600005 16386122

[98] Gat Y Bachar GN Everaert K Levinger U Gornish M. Induction of spermatogenesis in azoospermic men after internal spermatic vein embolization for the treatment of varicocele. Hum Reprod. (2005) 20:1013–7. 10.1093/humrep/deh706 15618245

[99] Poulakis V Ferakis N de Vries R Witzsch U Becht E. Induction of spermatogenesis in men with azoospermia or severe oligoteratoasthenospermia after antegrade internal spermatic vein sclerotherapy for the treatment of varicocele. Asian J Androl. (2006) 8:613–9. 10.1111/j.1745-7262.2006.00157.x 16847530

[100] Ishikawa T Kondo Y Yamaguchi K Sakamoto Y Fujisawa M. Effect of varicocelectomy on patients with unobstructive azoospermia and severe oligospermia. BJU Int. (2008) 101:216–8. 10.1111/j.1464-410X.2007.07279.x 17941933

[101] Youssef T Abd-Elaal E Gaballah G Elhanbly S Eldosoky E. Varicocelectomy in men with nonobstructive azoospermia: is it beneficial? Int J Surg. (2009) 7:356–60. 10.1016/j.ijsu.2009.05.009 19482096

[102] Cocuzza M Pagani R Lopes RI Athayde KS Lucon AM Srougi M. Use of subinguinal incision for microsurgical testicular biopsy during varicocelectomy in men with nonobstructive azoospermia. Fertil Steril. (2009) 91:925–8. 10.1016/j.fertnstert.2007.12.065 18644594

[103] Kirac M Deniz N Biri H. The effect of microsurgical varicocelectomy on semen parameters in men with non-obstructive azoospermia. Curr Urol. (2013) 6:136–40. 10.1159/000343527 24917731 PMC3783270

[104] D'Andrea S Giordano AV Carducci S Sacchetti L Necozione S Costanzo M. Embolization of left spermatic vein in non-obstructive azoospermic men with varicocele: role of FSH to predict the appearance of ejaculated spermatozoa after treatment. J Endocrinol Invest. (2015) 38:785–90. 10.1007/s40618-015-0259-x 25740066

[105] Elbardisi H El Ansari W Majzoub A Arafa M. Does varicocelectomy improve semen in men with azoospermia and clinically palpable varicocele? Andrologia. (2020) 52:e13486. 10.1111/and.13486 31825116

